# Enhanced Enzymatic Hydrolysis of Rice Straw Pretreated by Oxidants Assisted with Photocatalysis Technology

**DOI:** 10.3390/ma11050802

**Published:** 2018-05-16

**Authors:** Ken-Lin Chang, Xiao-Qin Wang, Ye-Ju Han, Hao Deng, Jing-yong Liu, Yuan-Chung Lin

**Affiliations:** 1 School of Environmental Science and Engineering and Institute of Environmental Health and Pollution Control, Guangdong University of Technology, Guangzhou 51006, China; klchang@mail.nsysu.edu.tw (K.-L.C.); gdut-xqwang@foxmail.com (X.-Q.W.); gduthyj@163.com (Y.-J.H.); lynxpatom@gmail.com (H.D.); www053991@126.com (J.-y.L.); 2 Institute of Environmental Engineering, National Sun Yat-Sen University, Kaohsiung 80424, Taiwan; 3 College of Pharmacy, Kaohsiung Medical University, Kaohsiung 000807, Taiwan

**Keywords:** photocatalysis, oxidants, pretreatment, enzymatic hydrolysis

## Abstract

This work evaluated the effectiveness of rice straw pretreatment using a TiO_2_/UV system in the presence of oxidants. The effects of TiO_2_ concentrations, pH and photocatalysis time were investigated. Inorganic oxidants including H_2_O_2,_ K_2_S_2_O_8_, and KIO_4_ were added to further enhance the effect on enzymatic hydrolysis of rice straw. The TiO_2_/UV/ H_2_O_2_ pretreatment showed a higher amount of released reducing sugar (8.88 ± 0.10 mg/mL, compared to 5.47 ± 0.03 mg/mL in untreated sample). Composition analyses of rice straw after the TiO_2_/UV/H_2_O_2_ pretreatment showed partial lignin and hemicellulose removal. Moreover, structural features of untreated and pretreated rice straw were analyzed through FE-SEM, FT-IR, and XRD. This work suggests that H_2_O_2_ is an efficient addition for photocatalysis pretreatment of rice straw.

## 1. Introduction

Lignocellulosic biomass is an abundant and sustainable energy resource for producing of biofuels and valuable chemicals, such as bioethanol, biobutanol, xylitol and lactic acid [1]. The use of lignocellulosic biomass as an alternative fuel is considered one of the best solutions to the current energy crisis. Furthermore, utilizing lignocellulosic biomass reduces greenhouse gas emissions, promotes rural area economic development, and enhances energy security [2,3]. Despite these advantages, the biomass recalcitrance due to the cuticle and epicuticular waxes, structural heterogeneity, degree of lignification and crystalline cellulose of lignocellulosic biomass is a challenge in the search to obtain high bioethanol production [4,5]. Herein, finding a pretreatment that can break down biomass recalcitrance is the imperative for all forms and types of lignocellulosic biomass [6,7].

A suitable pretreatment technology should be both financially viable and effective [8]. It could also facilitate lignin or hemicellulose removal, disrupt hydrogen bonds in crystalline cellulose and increase enzymatic access to cellulose [7,9]. Among several pretreatments, photocatalytic oxidation has attracted significant attention for the depolymerization of lignin under mild conditions [10]. For a potential photocatalyst, there has been increasing interest in environmental applications of titanium dioxide (TiO_2_) due to its commercial availability and chemical stability [11]. It is capable of indirect photolysis or heterogeneous photocatalysis when TiO_2_ combines with ultraviolet (UV) light [12]. It is generally accepted that OH radical oxidation and electron–hole reaction occur due to the short wavelength of UV light to complete the photolysis process [13], and show very fast reaction rates [14,15]. Several groups have used the pretreatment technology of TiO_2_/UV to degrade organic contaminants [16,17], and the TiO_2_/UV process can successfully pretreat paper mill effluent, black liquor, and olive mill waste water [10,18]. Moreover, some attempts have been made to depolymerize lignin with a simpler structure [11]. However, TiO_2_ as a photocatalyst produces hydroxyl radicals that cannot be infinitely increased, and adding an excess amount of titanium dioxide alone will disperse the ultraviolet light in the solution.

To reduce the probability of recombing photogenerated electrons and holes, more holes and increasing hydroxyl radical concentration can be provided for oxidative degradation of organic contaminants. Some researchers have found that the presence of oxidants can promote the performance of TiO_2_/UV process [19]. Gözmen [20] observed that increasing periodate ion concentration (1–5 mM) enhances the degradation of basic dyes in the TiO_2_/UV system. Inorganic oxidants such as IO_4_^−^, BrO_3_^−^, S_2_O_8_^2−^, and H_2_O_2_ can quench conduction band electrons and form reactive radical intermediates, so they are used as additives applied in TiO_2_/UV [19].

Photocatalysis, are considered to have mild pretreatment conditions and provide an environmentally friendly alternative. The TiO_2_/UV process has been successfully applied to pretreat organic species [10]. Related research has focused mainly on using the TiO_2_/UV system to degrade commercial lignin powder and lignin solutions [11]. Compared with native biomass, commercial lignin has a less complicated structure. To the best of our knowledge, there have been very few studies based on native biomass, such as rice straw. This study utilized oxidants such as H_2_O_2_, K_2_S_2_O_8_ and KIO_4_ as additives in a TiO_2_/UV system. They have high polarizability so that they can capture the electrons ejected from TiO_2_ [21] and are more receptive to price. This study: (i) investigated the effects of TiO_2_ concentrations, pH, and photocatalysis time; (ii) determined the effectiveness of oxidants such as KIO_4_, K_2_S_2_O_8_ and H_2_O_2_ as additives for rice straw pretreatment with a TiO_2_/UV system; (iii) investigated oxidants can improve the pretreatment efficacy and how they function during the process; and (iv) investigated the optimal oxidant to achieve the desirable enzymatic hydrolysis efficiency.

## 2. Materials and Methods

### 2.1. Materials and Chemical Reagents

Rice straw was supplied by the Chinese Academy of Sciences (Xiamen, China). It was cut into small pieces, dried at 60 °C, and then ground and screened to 40 mesh size particles for further use. TiO_2_ (99.9%) was purchased from Sigma Aldrich Co., Ltd. (St. Louis, MO, USA) The photocatalytic device was purchased from BiLon Co., Ltd. (Xian, China) The UV lamp (36W, BiLon Co., Ltd., Xian, China) with irradiation wavelength 254 nm. KIO_4_, K_2_S_2_O_8_, and H_2_O_2_ [30% (*w*/*w*)] were obtained from Guangzhou Chemical Reagent Factory (Guangzhou, China). All other reagents were analytical grade.

### 2.2. Photocatalysis Pretreatment

The rice straw powder was first dewaxed in acetone at 20 °C for 16 h [22]. The dewaxed rice straw was cleaned with deionized water and oven dried at 60 °C for 24 h. Sample preparation consisted of soaking 1.0 g rice straw in 40 mL TiO_2_ solution. The initial pH (3, 4, 6, 7, 8, and 10) was adjusted by addition of appropriate amounts of HCl (1N) or NaOH (1N). TiO_2_ concentrations were 0%, 0.05%, 0.25%, 0.50%, 1.0%, and 2.0% (*w*/*v*). The sample preparation was transferred into a 500-mL beaker and exposed to UV irradiation in a device at ambient temperature (30 ± 1 °C), with a magnetic stirrer. The UV lamp was positioned in a quartz glass vessel to maintain a uniform distribution of UV light during pretreatment. The UV lamp was in a fixed position in the center of the bottom of the beaker. UV lamp irradiation times ranged from 0 to 8 h (i.e., 0, 1, 2, 3, 4, 6 and 8 h).

### 2.3. Enzymatic Hydrolysis of Rice Straw

Untreated and pretreated samples were tested at a solid loading of 2.5% (*w*/*v*) in 25-mL conical flasks. Each sample enzymatic hydrolysis reaction contained 0.05 M sodium citrate buffer (pH = 4.8), 0.02% sodium azide, cellulase complex (Novozyme NS220086, 250 FPU mL^−1^, Copenhagen, Denmark) at a loading of 50 FPU/g and β-glucosidase (Novozyme NS221118, 320 CBU mL^−1^) at a loading of 40 CBU/g. The reactions were carried out in a horizontal shaker incubator (150 rpm, Jinghong Experimental Equipment Co., Ltd., Shanghai, China) for 48 h at 50 °C. Samples taken after 0 and 48 h of hydrolysis and centrifuged to remove the residue at 7000 rpm were analyzed for released reducing sugar by the dinitrosalicylic acid (DNS) method [23].

### 2.4. Addition of Oxidants in a TiO_2_/UV System

For experiments with TiO_2_/UV/oxidants, TiO_2_ concentrations were 0.50% (*w*/*v*). Three types of oxidants were used only for rice straw pretreatment in the TiO_2_/UV system: KIO_4_, K_2_S_2_O_8_, and H_2_O_2_ (1–16 mM). The UV irradiation time was 3 h.

### 2.5. Characterization Analyses

The morphologies of untreated and pretreated rice straw were performed using JSM-7001F Field-Emission Scanning Electron Microscope (JEOL, Tokyo, Japan). The samples were fixed with conductive tape on brass sample stubs and then coated with Au before observation. Images were acquired at a 15-KV acceleration voltage and magnification × 500. 

The untreated and pretreated samples for Fourier Transform Infrared (FTIR) spectroscopic analysis were determined using a Nicolet 6700 spectrometer (Thermo Fisher Scientific, Waltham, MA, USA) over a wavenumber range from 500–4000 cm^−1^ with 20 scans and a resolution of 4 cm^−1^. The samples were dispersed of spectroscopic grade potassium bromide (KBr) and pressed into disks to determined.

X-ray diffraction analysis was tested using an Ultima X-ray diffractometer of Rigaku Corporation (40 KV, 20 mA, Tokyo, Japan) with Cu Kα radiation. The 2θ range was from 5° to 40° in steps of 0.02° at a rate of 8° min^−1^ [24]. The crystallinity index (CrI) of untreated and pretreated samples was calculated according to Equation (1) [25].

(1) $$\mathrm{CrI}\%=\frac{I_{002}-I_{am}}{I_{002}}\times 100\%$$

where $I_{002}$ is the intensity of the crystalline regions (2θ = 22.5°) and $I_{am}$ is the intensity of amorphous portion (2θ = 18.7°).

## 3. Results

### 3.1. Optimization of Conditions for Photocatalysis Pretreatment

**Figure 1:**
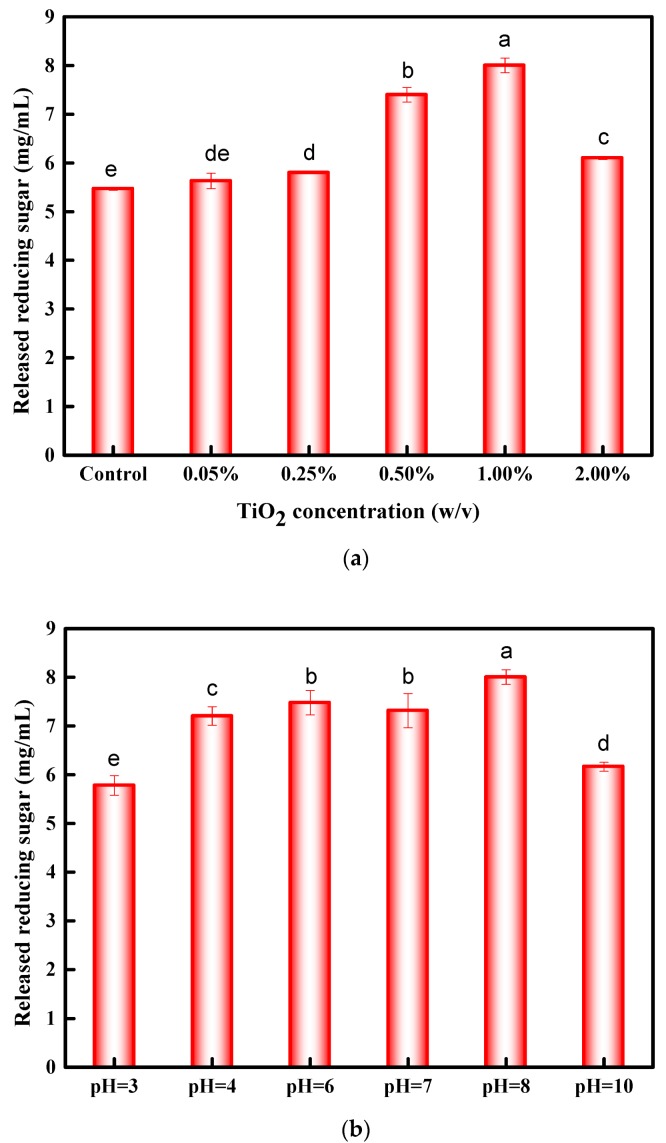

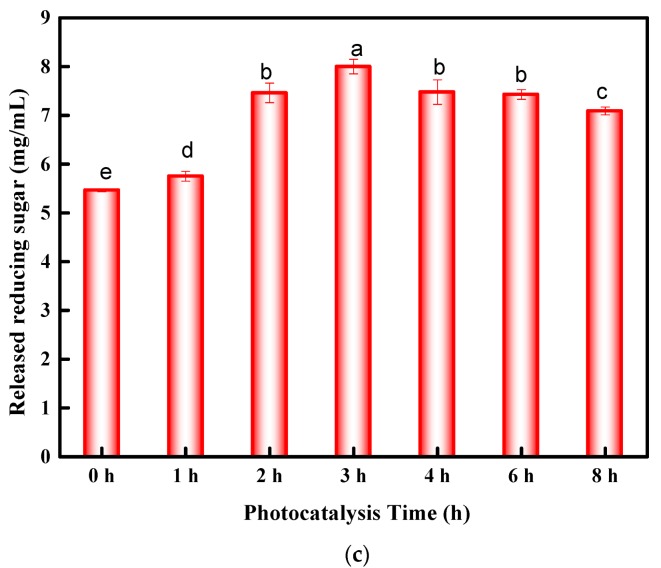
(**a**) Effect of TiO_2_ concentration on enzymatic hydrolysis of rice straw; (**b**) effect of pH on enzymatic hydrolysis of rice straw; and (**c**) effect of photocatalysis time on enzymatic hydrolysis of rice straw. The different letters above the bars in figures indicate significantly difference (*p* ≤ 0.05, n = 3).

The variables used in the pretreatment were TiO_2_ concentrations, pH solution, and photocatalysis irradiation time. To optimize pretreatment conditions, the selected TiO_2_ concentrations were 0%, 0.05%, 0.25%, 0.50%, 1.0%, and 2.0% (*w*/*v*). The effect of TiO_2_ concentration on enzymatic hydrolysis of rice straw is illustrated in Figure 1a, in which the released amount of reducing sugar increased with the TiO_2_ concentration from 0.05% to 1.0% (*w*/*v*). The highest released reducing sugar was 8.04 ± 0.15 mg/mL, observed at TiO_2_ concentration of 1.0% (*w*/*v*). Higher TiO_2_ concentration absorbed more energy during UV irradiation process. However, adding an excess amount of TiO_2_ alone will disperse the ultraviolet light in the solution, markedly decreasing the enzymatic hydrolysis efficiency. Consequently, optimal TiO_2_ concentration was 1.0% (*w*/*v*).

The solution pH has a significant effect on photocatalysis reactions taking place on the TiO_2_ surface. Thus, the pH of solution was studied for enzymatic hydrolysis of rice straw in the pH range of 3–10 at a fixed concentration of TiO_2_ (1.0% (*w*/*v*)). Mechanisms of the TiO_2_/UV process on the effect of pH are complex and the solution pH dictates the surface charge properties of the photocatalyst [26]. TiO_2_ is an amphoteric metal oxide, and, at more acidic values, the surface will be positively charged. Conversely, the surface is negatively charged. A pH that is too high or too low is not suitable for TiO_2_/UV on enzymatic hydrolysis of rice straw, as shown in Figure 1b. The pH value of 8 was selected as the most suitable photocatalysis pretreatment.

Photocatalysis times and their effects on enzymatic hydrolysis of rice straw were investigated. Figure 1c shows that the released reducing sugar increased when irradiation time ranged from 0 to 8 h. The TiO_2_/UV pretreatment time was prolonged from 1 to 3 h. As the irradiation time increased to 8 h, enzymatic hydrolysis of rice straw was reduced. Therefore, optimal conditions for the TiO_2_/UV pretreatment were 3 h, pH 8, and TiO_2_ concentration 1.0% (*w*/*v*).

### 3.2. Effect of Added Oxidants in TiO_2_/UV Pretreatment on Enzymatic Hydrolysis of Rice Straw

**Figure 2:**
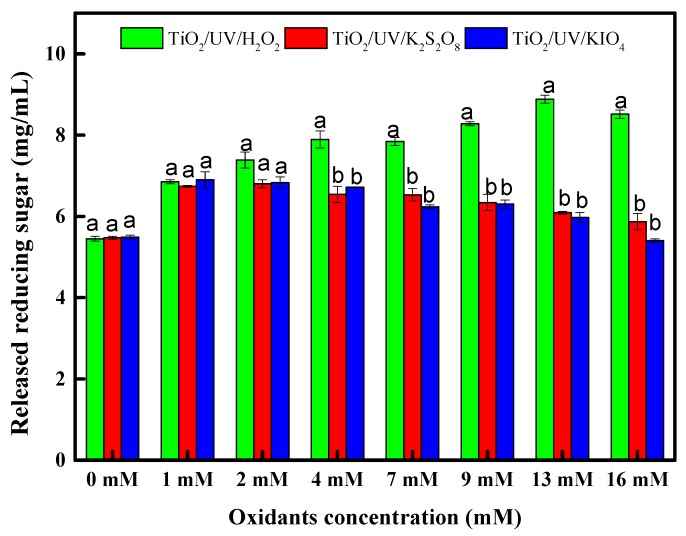
Effect of added different oxidants concentration in TiO_2_/UV pretreatment on enzymatic hydrolysis of rice straw. The different letters above the bars indicate significant difference (*p* ≤ 0.05, n = 3).

In subsequent experiments, the reducing dosage of TiO_2_ was chosen as a concentration of 0.50% (*w*/*v*). The added oxidants in a TiO_2_/UV system function as electronic scavengers, as well as the oxidant itself. The released reducing sugar was 5.47 ± 0.03 mg/mL of TiO_2_/UV pretreatment, for which TiO_2_ concentration was 0.50% (*w*/*v*) without oxidant added. As indicated in Figure 2, adding inorganic oxidants increased the released reducing sugar of rice straw in the TiO_2_/UV system. The optimal released reducing sugar for TiO_2_/UV/K_2_S_2_O_8_, TiO_2_/UV/KIO_4_ and TiO_2_/UV/H_2_O_2_ pretreatments were 6.80 ± 0.10 mg/mL, 6.90 ± 0.20 mg/mL and 8.88 ± 0.10 mg/mL, respectively. These results confirm previous reports that TiO_2_/UV/oxidant provided better photocatalysis than TiO_2_/UV alone. For increasing hydrolysis, H_2_O_2_ was more effective than K_2_S_2_O_8_ or KIO_4_, and excess K_2_S_2_O_8_ and KIO_4_ reduced the hydrolysis efficiency of rice straw in a TO_2_/UV system. The excess of K_2_S_2_O_8_ inhibited photocatalysis process, due to the increase of sulfate radical anion in solution, was adsorbed on the TiO_2_ surface. The adsorbed sulfate radical anion also reacted with hydroxyl radicals, thus reducing the photocatalytic activity [27]. Further increase of KIO_4_ decreased the photocatalytic efficiency, as the excess periodate ion in solution may scavenge hydroxyl radicals [19]. Released reducing sugar increased with increasing H_2_O_2_ concentration up to 13 mM, but exceeding 13 mM of H_2_O_2_ concentration decreased the hydrolysis efficiency. In particular, the addition of 13 mM H_2_O_2_ had a greater impact than the TiO_2_ concentration of 0.50% in the TiO_2_/UV system. The released reducing sugar from TiO_2_/UV/H_2_O_2_ pretreatment was greater than TiO_2_/UV/K_2_S_2_O_8_ and TiO_2_/UV/KIO_4_ pretreatment. The addition of oxidants in a TiO_2_/UV system are generally applied for treating reactive dyes and organic contaminants in industrial wastewater [19,28]. Prior studies have proposed that the role of oxidants in TiO_2_/UV system increased the formation of hydroxyl radicals and impeded electron–hole recombination. Hence, a proper concentration of H_2_O_2_ was important for enzymatic hydrolysis of rice straw.

### 3.3. Composition Analysis of Untreated and Pretreated Rice Straw

Table 1 summarizes the compositions of untreated and pretreated rice straw. Optimum conditions were chosen for the three oxidants to analyze chemical compositions and selected the corresponding control group of oxidants for comparison. The contents of untreated rice straw were determined as lignin (18.68 ± 0.30%), hemicellulose (32.98 ± 1.10%), and cellulose (37.47 ± 0.57%), similar to previous studies [29,30]. The H_2_O_2_ alone, H_2_O_2_/UV pretreatment and TiO_2_/UV/H_2_O_2_ pretreatment of lignin contents were 17.04 ± 0.74%, 17.45 ± 0.99% and 16.10 ± 0.67%, respectively. Lignin levels of TiO_2_/UV/K_2_S_2_O_8_ and TiO_2_/UV/KIO_4_ pretreatments were 18.41 ± 0.33% and 17.69 ± 0.54%, respectively. This demonstrated that the TiO_2_/UV/H_2_O_2_ pretreatment improved enzymatic hydrolysis of rice straw. Some research has proposed lignin removal as an essential factor for enzymatic saccharification of lignocellulosic biomasses [31]. Hemicellulose contents by TiO_2_/UV/K_2_S_2_O_8_ and TiO_2_/UV/KIO_4_ pretreatment were 31.46 ± 0.28% and 32.63 ± 0.31% compared with 32.98 ± 1.10% for the untreated. Öhgren [32] reported that hemicellulose hinders enzyme access to cellulose, reducing the cellulase accessibility of lignocellulose biomass. As noted above, the components of rice straw after TiO_2_/UV/K_2_S_2_O_8_ and TiO_2_/UV/KIO_4_ pretreatment showed no significant difference in the content of lignin, cellulose and hemicellulose. Composition analysis demonstrated that TiO_2_/UV/H_2_O_2_ pretreatment improved enzymatic efficiency more than pretreatment with other oxidants to support TiO_2_/UV.

### 3.4. FE-SEM, XRD and FTIR Profile of Untreated and Pretreated Rice Straw

**Figure 3:**
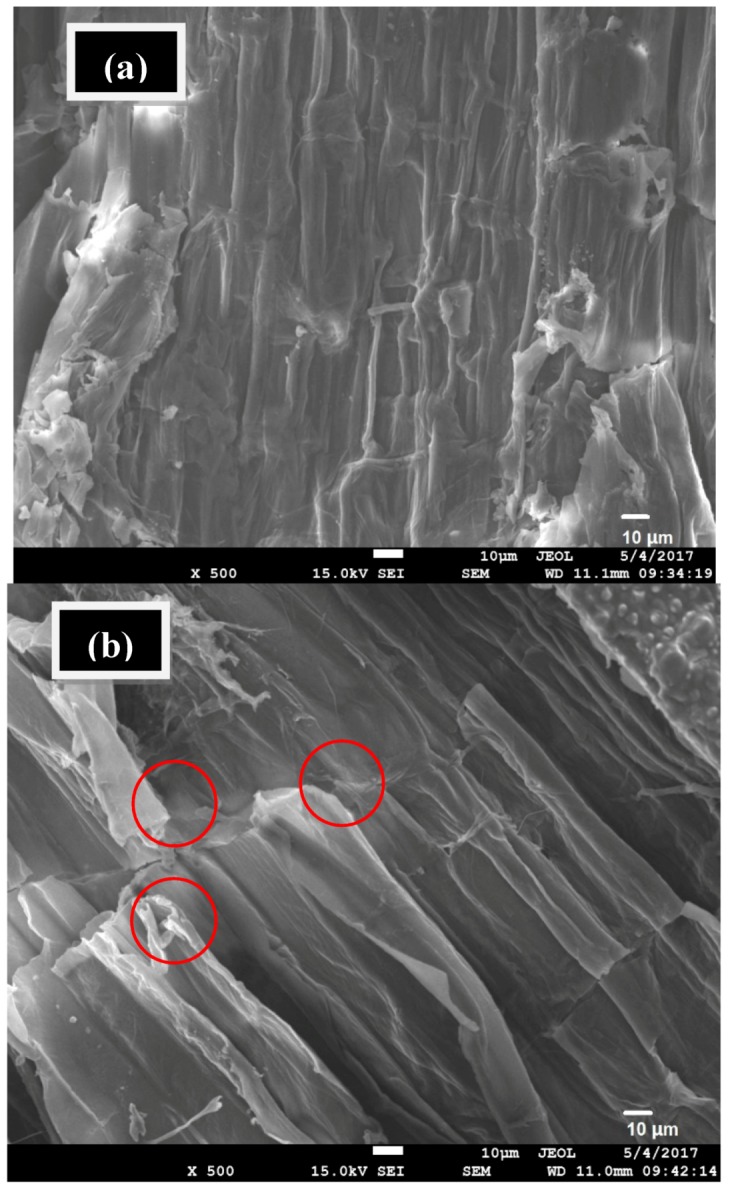
(**a**) FE-SEM images of untreated rice straw; and (**b**) FE-SEM images of TiO_2_/UV/H_2_O_2_ pretreatment of 13 mM H_2_O_2_ at the condition of pH = 4, irradiation time of 3 h and 0.50% (*w*/*v*) concentration of TiO_2_.

**Figure 4:**
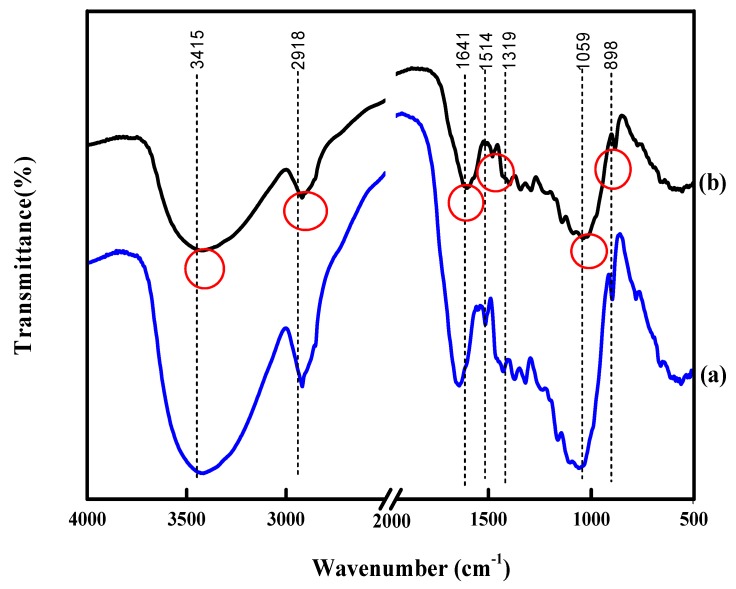
FT-IR images of untreated rice straw (**a**) and TiO_2_/UV/H_2_O_2_ pretreatment (**b**) at the condition of pH = 4, irradiation time of 3 h and 0.50% (*w/v*) concentration of TiO_2_.

FE-SEM was performed to observe the morphological structures of untreated and pretreated rice straw and determine structural changes of the sample surfaces after photocatalysis pretreatment. Untreated rice straw (Figure 3a) presented flat and well-ordered surface structure. FE-SEM images show that the TiO_2_/UV/H_2_O_2_ pretreated sample (Figure 3b) has a different morphology, disrupting the extensive, well-ordered surface of rice straw. Moreover, the surface has loose and irregular structure, with some cracks on the surface (marked with red circle). Changes of pretreated rice straw were beneficial to the enzymatic hydrolysis process due to the enhancement of cellulose accessibility to the enzyme. FTIR spectroscopy identified the changes of organic groups of untreated and pretreated rice straw, as shown in Figure 4 (marked with red circle). An untreated sample of rice straw was observed and compared with a pretreated sample obtained at the conditions of TiO_2_/UV/H_2_O_2_. The band at 3415 cm^−1^ was subject to O–H stretching of the hydroxyl group. Among the peaks, 2918 cm^−1^ (C–H stretching of methyl and methyene groups), 1059 cm^−1^ (C–O stretching vibration) and 898 cm^−1^ (C–H deformation vibration) were assigned as the characteristic peaks of cellulose and hemicellulose. A decrease was observed in the absorbance of the 1641 cm^−1^ of conjugated carbonyl stretch in lignin [33]. Moreover, lignin-related peaks had some changes in the vibration at the absorbance bands of 1514 cm^−1^ (aromatic skeletal vibrations) and 1319 cm^−1^ (C–O vibration in the syringyl ring). With TiO_2_/UV/H_2_O_2_, the rice straw was weaker than the untreated sample, indicating greater lignin removal.

**Figure 5:**
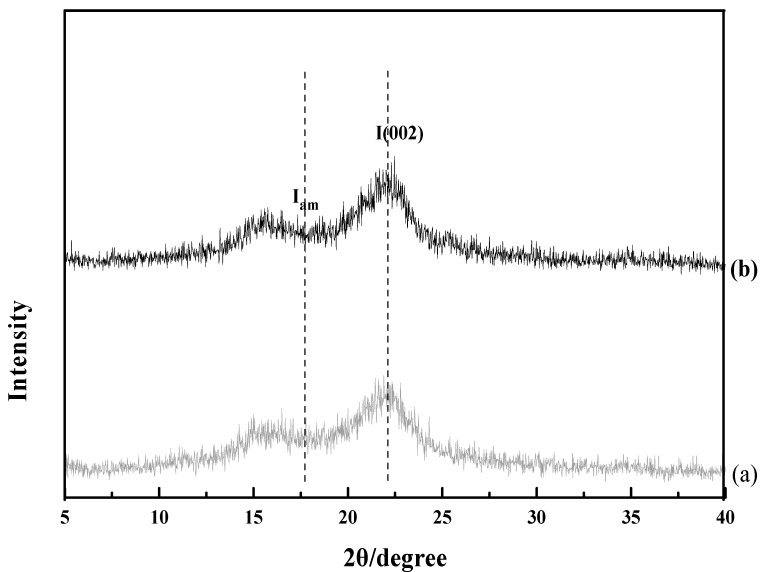
XRD analysis of untreated rice straw (**a**) and TiO_2_/UV/H_2_O_2_ pretreatment (**b**) at the condition of pH = 4, irradiation time 3 h and 0.50% (*w*/*v*) concentration of TiO_2_.

Figure 5 shows XRD diffraction of untreated and TiO_2_/UV/H_2_O_2_ pretreated rice straw. The CrI of untreated and TiO_2_/UV/H_2_O_2_ pretreatment rice straw was calculated according to Equation (1). The CrI of untreated sample was 43.90%, and the CrI of TiO_2_/UV/H_2_O_2_ pretreatment was 46.63%. TiO_2_/UV/H_2_O_2_ pretreatment increased the intensity of the removal of amorphous components such as hemicellulose and lignin. Additionally, this pretreatment influenced enzymatic hydrolysis that released reducing sugar. There were similar observations of increased crystallinity index for biomass pretreatments such as previous surfactant-assisted ultrasound-ionic liquid-based pretreatment and concentrated phosphoric acid plus hydrogen peroxide pretreatment, where the crystallinity index increased by deconstruction of the crystalline cellulose [34,35].

## 4. Conclusions

This work suggests adding oxidants can boost lignin removal and improve the release of reducing sugar of rice straw. Among the oxidants, H_2_O_2_ addition in the TiO_2_/UV system for pretreated rice straw was most effective to reduce the dosage of TiO_2_ under the same pretreatment conditions. Optimal TiO_2_/UV/H_2_O_2_ pretreatment conditions for rice straw were adding 13 mM H_2_O_2_ at pH = 4, irradiation time of 3 h and 0.50% (*w*/*v*) concentration of TiO_2_. Results of the composition and characterization analyses indicated that TiO_2_/UV/H_2_O_2_ pretreatment efficiently provided lignin removal and significant changes in morphology.

## Figures and Tables

**Table 1:** Chemical composition of untreated and pretreated rice straw.

Pretreatment	Cellulose (%)	Hemicellulose (%)	Lignin (%)
Untreated	37.47 ± 0.57	32.98 ± 1.10	18.68 ± 0.30
H_2_O_2_	39.68 ± 0.64	32.49 ± 0.62	17.04 ± 0.74
H_2_O_2_/UV	40.59 ± 1.27	32.16 ± 1.14	17.45 ± 0.99
TiO_2_/UV/H_2_O_2_	42.00 ± 0.70	30.14 ± 1.12	16.10 ± 0.67
K_2_S_2_O_8_	40.21 ± 0.15	31.04 ± 0.81	18.12 ± 0.74
UV/K_2_S_2_O_8_	40.58 ± 0.39	31.05 ± 1.40	18.70 ± 0.10
TiO_2_/UV/K_2_S_2_O_8_	40.77 ± 0.66	31.46 ± 0.28	18.41 ± 0.33
KIO_4_	40.21 ± 0.81	31.90 ± 0.60	17.75 ± 0.23
UV/KIO_4_	41.81 ± 0.10	31.76 ± 0.25	18.05 ± 0.59
TiO_2_/UV/KIO_4_	39.72 ± 0.69	32.63 ± 0.31	17.69 ± 0.54
